# From Defenders to Hosts: The Arm-in-Arm Expansion of KRAB-Zinc Finger Gene Repertoires and Retroviral Integrations in Mammals

**DOI:** 10.3390/v18101153

**Published:** 2026-10-17

**Authors:** Melania Bruno

**Affiliations:** Independent Researcher, 80053 Castellammare di Stabia, Italy; micro.melania.bruno@gmail.com

**Keywords:** transposable elements, endogenous retroviruses, KRAB-zinc finger proteins, repetitive gene clusters

## Abstract

KRAB-zinc finger proteins (KZFPs) constitute one of the largest and most rapidly evolving families of transcription factors in vertebrates, with particularly extensive expansion and diversification in mammals. While several KZFPs are conserved across species, numerous lineage- and species-specific genes have emerged during mammalian evolution, with a large fraction recognizing and repressing transposable elements (TEs), and particularly endogenous retroviruses (ERVs). The evolutionary relationship between KZFPs and ERVs has traditionally been interpreted through an “arms-race” model, in which recurrent retroviral invasions drive the emergence of new KZFP specificities, while retroelements evolve to escape host repression. This review examines the evidence linking the evolutionary dynamics of KZFPs and ERVs and discusses how their relationship may extend beyond reciprocal adaptation at the level of sequence recognition. In particular, recent comparative genomic analyses reveal that young KZFP genes are frequently organized in highly repetitive genomic clusters that are themselves enriched in young ERVs. Structural variation and segmental duplication within these regions can simultaneously expand KZFP and ERV copy numbers, providing a potential genomic mechanism for their concerted diversification. These observations motivate an “arm-in-arm” model, in which KZFPs and ERVs are not only antagonistic evolutionary partners but can also become physically and evolutionarily intertwined within the same genomic environments. The possibility that KZFP clusters may have a dual role in the evolutionary fate of retroviral sequences is also considered: while KZFP-mediated repression can limit the deleterious effects of newly integrated retroelements and facilitate their persistence, the repetitive and structurally dynamic nature of these loci may create opportunities for subsequent duplication and diversification. Together, these observations broaden the classical arms-race framework and highlight how the interplay between retroviral invasion, host defense, genomic architecture, and sequence cooption can contribute to the generation of genomic and regulatory diversity.

## 1. Introduction

Among the molecular forces shaping genome evolution, transposable elements (TEs) represent one of the most prominent sources of genetic variation, contributing to sequence and, in some cases, phenotypic diversity within and between species [1,2]. Once only considered parasitic and selfish genetic elements that propagate at the expense of their hosts, TEs are increasingly recognized for their contributions to host genome evolution and biology [3,4].

Different TE classes have contributed to host evolution through various mechanisms [5]. Among them, long terminal repeat (LTR) retrotransposons, and particularly endogenous retroviruses (ERVs), represent perhaps the most striking example of how genomic parasites can become sources of host innovation. ERVs indeed originate from ancient retroviral infections that became fixed in the germline and were subsequently inherited across generations, becoming part of the host genome [6]. While uncontrolled ERV activity poses a threat to genome integrity through insertional mutagenesis, recombination, and immune activation [7,8], a growing body of evidence demonstrates that both protein-coding and regulatory sequences derived from ERVs have been repeatedly coopted for host functions [9,10]. The evolutionary integration of these retroviral-derived sequences into host biology has therefore required regulatory mechanisms capable of restricting their harmful activities while permitting their controlled expression in specific developmental and physiological contexts [11].

Among these regulatory systems, KRAB-zinc finger proteins (KZFPs) represent the best characterized family of transcription factors that mediate sequence-specific repression of transposable elements, including a large fraction of ERVs [12]. Beyond their canonical role in ERV silencing, KZFPs are increasingly recognized as transcriptional regulators that facilitate the incorporation of ERV-derived sequences into host gene regulatory networks [13].

In this review, we first summarize some representative examples of TE and ERV cooption before discussing how KZFP repertoires and endogenous retroviral sequences have been coevolving in mammals. Building on recent evidence, we propose an “arm-in-arm” model in which the expansion of KZFPs and ERVs reflects not only evolutionary conflict, but also a process of mutual diversification that has generated new regulatory functions and contributed to genome innovation.

## 2. From Genomic Parasites to Sources of Host Innovation: TE Co-Option and Domestication

The classical view of transposable elements (TEs) as selfish DNA emphasizes the conflict between mobile elements and their hosts. Yet, host genomes are not merely passive substrates in this evolutionary conflict. Rather, they are active participants in genome evolution, repeatedly capturing and repurposing TE sequences when their integration is either selectively neutral or, in some cases, confers a fitness advantage. The growing catalog of TE co-option events illustrates that all major TE classes have contributed to host genome evolution and biology [5,14].

DNA transposons, which mobilize through a cut-and-paste mechanism when active, have repeatedly contributed protein domains that have been domesticated by host genomes, generating novel cellular functions [15,16,17,18]. Retrotransposons, which amplify through copy-and-paste mechanisms, have similarly provided abundant sources of genetic innovation both as protein-coding sequences and as regulatory elements that contribute to gene regulatory networks. Short interspersed nuclear elements (SINEs) have been shown to contribute to several layers of genome function, from three-dimensional genome organization [19,20] to transcription regulation and alternative splicing [21,22,23,24,25]. Long interspersed nuclear elements (LINEs), particularly LINE-1 elements, have likewise contributed promoters, enhancers, and other regulatory sequences [26,27,28,29,30].

Among retrotransposons, long terminal repeat (LTR) retrotransposons, and particularly endogenous retroviruses (ERVs), represent perhaps the most striking example of the evolutionary transition from genomic parasites to sources of host innovation. ERVs originate from ancient retroviral infections that became fixed in the germline and were subsequently inherited across generations. Although their viral origin makes them potential sources of genomic instability and immune activation, ERV-derived sequences have repeatedly been coopted by mammalian genomes at both the protein and regulatory levels. Domesticated genes derived from LTR retroelements have acquired essential physiological functions in diverse biological contexts, including ARC, important for synaptic plasticity and neuronal communication [31], PEG10 and syncytins, which are indispensable for mammalian placentation [32,33,34], as well as Suppressyn, an ERV envelope-derived protein that protects human cells against retroviral infection [35]. Beyond protein-coding genes, ERV-derived regulatory sequences have been extensively incorporated into host gene regulatory networks, contributing to processes ranging from innate immune responses to early embryonic development and zygotic genome activation [9,36,37,38].

The successful co-option of retroviral sequences nevertheless requires an evolutionary balance [39]. On the one hand, ERV activity can be detrimental to the host through effects on genome stability and the activation of innate immune and inflammatory pathways. ERV insertions can act as genomic mutagens and substrates for non-allelic homologous recombination, while derepression of ERV loci can generate viral-like nucleic acids that activate innate immune sensing pathways [40,41,42,43]. Indeed, aberrant ERV activity has been associated with inflammatory processes in contexts such as cancer and cellular senescence, while individual ERV integrations can impair physiology or cause specific developmental defects [44,45,46,47,48]. Accordingly, organisms have evolved multiple mechanisms to detect and repress these elements, reflecting a long-standing conflict between host genomes and their genomic parasites [49,50].

At the same time, repression of potentially harmful retroviral sequences may allow them to persist within host genomes, creating an opportunity for their subsequent co-option. The importance of this balance is particularly evident during ongoing retroviral endogenization. The koala retrovirus (KoRV), for example, provides a rare opportunity to observe the early stages of this process in real time. KoRV has been undergoing germline invasion in koala populations, with endogenous integrations coexisting with actively transmitted virus, providing a snapshot of a retrovirus in transition from an exogenous pathogen to a heritable genomic element [51]. More recent genomic studies have revealed extensive variation in KoRV integration sites and recombinant forms generated during this ongoing germline invasion [52,53]. Thus, the evolutionary trajectory of retroviral sequences does not necessarily end with their elimination or complete inactivation: some may persist under host repression and, over evolutionary time, acquire functions that can be integrated into host biology.

The evolutionary integration of ERV-derived sequences into host biology therefore requires regulatory systems capable of modulating their activity in a context-dependent manner. Successful ERV domestication relies not only on the broad repression of potentially harmful retroviral activities, but also on the fine-tuned regulation of individual ERV-derived loci as functional components of host gene regulatory networks. This dual requirement creates a central evolutionary tension: the host must control the potentially deleterious activities of ERVs while retaining, and in some cases exploiting, the genetic and regulatory potential embedded within these sequences. Among the molecular systems that have evolved at this interface, KRAB-zinc finger proteins (KZFPs) occupy a particularly prominent position [54].

## 3. KRAB-Zinc Finger Proteins at the Host–Retroelement Interface

KZFPs represent the best example we have thus far of transcription factors that can act both as repressors of TE activity, as well as regulators that can facilitate the embedding of retroelement-derived sequences into host gene regulatory networks [12]. While different members of the KZFP family have been found to specifically repress distinct TE families belonging to any class, their ability to rapidly evolve as new ERV repressors makes them particularly intriguing from an evolutionary perspective.

### 3.1. KZFP Structure and Function

KZFPs are characterized by the combination of an N-terminal Krüppel-associated box (KRAB) domain and a C-terminal array of tandem C2H2 zinc fingers. Together, these domains can enable sequence-specific DNA recognition and transcriptional regulation, making KZFPs one of the largest families of transcriptional regulators in tetrapod genomes [54].

The C2H2 zinc finger is an evolutionarily ancient DNA-binding module composed of approximately 28 amino acids arranged in a conserved ββα fold stabilized by coordination of a zinc ion through two cysteine and two histidine residues [55]. DNA recognition is primarily mediated by residues within the α-helix, often referred to as the fingerprint residues, which contact bases in the DNA major groove. Individual zinc fingers typically recognize approximately three nucleotides, whereas tandem arrays—often comprising a dozen or more fingers—combine to generate the overall binding specificity of individual KZFPs, allowing recognition of relatively long genomic target sequences [56,57]. However, DNA-binding specificity cannot be fully inferred from the sequence of individual zinc fingers alone. Interactions between neighboring fingers, contacts with the DNA backbone, recognition of DNA shape, and sensitivity to epigenetic features such as DNA methylation can all contribute to target recognition [58]. Structural studies have further shown that not every zinc finger within an array participates directly in sequence recognition, with some instead contributing to protein-DNA or intramolecular interactions [59].

The KRAB domain is the defining effector module of the family and, in most KZFPs, functions as a potent transcriptional repression domain [60,61,62]. Although it lacks intrinsic enzymatic activity, its conserved A-box can recruit the KRAB-associated protein 1 (KAP1; also known as TRIM28 or TIF1β), thereby coupling sequence-specific DNA binding to epigenetic silencing [63,64]. KAP1 acts as a molecular scaffold that assembles a repressive chromatin complex including the histone methyltransferase SETDB1, heterochromatin protein 1 (HP1), and the NuRD chromatin-remodeling and histone deacetylase complex [65,66]. Through these interactions, KAP1 promotes H3K9me3 deposition, chromatin compaction, and transcriptional repression. However, not all KRAB domains interact with KAP1, indicating that KAP1 recruitment is not a universal property of KZFPs [67].

Although the canonical KZFP architecture consists of a KRAB domain linked to a zinc finger array, a subset of evolutionarily older KZFPs also contains additional N-terminal domains, most notably SCAN and DUF3669 [68]. Both domains mediate homo- and hetero-oligomerization between KZFPs carrying the corresponding accessory domain. Intriguingly, phylogenetic analyses indicate that both accessory domains themselves originated through transposable element domestication: the SCAN domain was derived from the capture of a Gypsy-like retrotransposon capsid protein [69], whereas recent evidence suggests that DUF3669 evolved from an L1-derived protein domain [70]. These findings illustrate that TEs have shaped not only the genomic targets of KZFPs, but also the evolution of the protein architecture itself in a subset of KZFPs.

Although KAP1-mediated transcriptional repression represents the best-characterized molecular mechanism of many KZFPs, some members of the family fulfill different biological functions. For example, the ancient KZFP PRDM9—the ancestor of the KZFP family—directs meiotic recombination hotspot specification through its PR/SET histone methyltransferase domain [71,72], whereas ZFP57 and ZFP445 are important regulators of genomic imprinting [73,74]. Other KZFPs have been implicated in developmental gene expression, cellular differentiation, and genome organization, illustrating how the conserved modular architecture of the family has supported extensive functional diversification during vertebrate evolution [75,76,77,78].

Nevertheless, a substantial fraction of mammalian KZFPs characterized to date have been associated with the recognition and regulation of TE-derived sequences, making this functional interaction a major force shaping the evolution of both families [12,79,80,81].

### 3.2. Functional Co-Evolution of KZFPs and ERVs: The Arms-Race Model

Since their emergence in the common ancestor of coelacanths and tetrapods more than 400 million years ago, KZFPs have undergone extensive expansion and diversification, particularly in the mammalian lineage, where they comprise one of the largest families of transcriptional regulators, with a few hundred members on average in mammalian genomes [12]. Although the overall number of KZFP genes is broadly comparable across many mammalian genomes, their repertoires are remarkably heterogeneous. Lineages can differ substantially in the identity and evolutionary age of their KZFPs, with particularly pronounced differences in lineage-, species-, and even subspecies-specific expansions, as observed when comparing primates and rodents (Figure 1) [12,81].

The observation that many evolutionarily young and lineage-restricted KZFPs repress TEs, and particularly ERVs, of similar age led to the proposal that the diversification of KZFP repertoires is driven by an evolutionary arms race with these genomic parasites. Several studies have revealed a striking correspondence between the evolutionary age of KZFPs and that of the ERVs they recognize, with younger KZFPs preferentially targeting evolutionarily young retroelements [12,79,80,81]. At the genomic level, this relationship is mirrored by a correlation between the abundance of LTR retroelements and tandem zinc-finger genes across vertebrate genomes, including a particularly strong association between recently active LTR elements and recently duplicated zinc-finger genes [82]. Together, these observations suggest that the repeated emergence of new retroelements may have provided selective pressure for the expansion and diversification of KZFP repertoires (Figure 1) [83].

Evidence for reciprocal adaptation has also emerged from individual KZFP-TE pairs. Several transposable elements have accumulated mutations that reduce or abolish recognition by existing KZFPs, followed in some cases by the emergence or diversification of KZFPs capable of recognizing the resulting escape variants. One of the clearest examples comes from the primate-specific KZFPs ZNF91 and ZNF93 [84]. ZNF91 underwent structural and sequence changes that enabled recognition and repression of SVA retrotransposons, whereas an ancestral LINE-1 lineage targeted by ZNF93 subsequently escaped repression through deletion of the ZNF93 recognition site.

These observations form the basis of the classical arms-race model, in which the emergence of new retroelements is counteracted by the evolution or diversification of KZFPs with new DNA-binding specificities. Conversely, sequence changes in retroelements can compromise existing repression and generate new selective pressure on the KZFP repertoire. The rapid evolution of both partners can therefore be interpreted as the product of reciprocal antagonistic interactions: retroelements evolve to escape host repression, whereas KZFPs evolve to restore control.

The arms-race model nevertheless does not fully capture the evolutionary relationship between KZFPs and their targets. Many KZFPs recognize TE-derived sequences that have lost transposition competence [12], raising the possibility that their continued repression reflects the regulatory activity of TE-derived sequences rather than the need to prevent further TE propagation. In this context, TE domestication provides an alternative evolutionary trajectory in which host genomes retain and repurpose TE-derived sequences while maintaining regulatory control over their activity. The persistence of KZFPs under purifying selection despite the loss of mobility of their target TEs therefore suggests that KZFP-mediated repression can remain functionally important after the original selective pressure imposed by TE transposition has diminished. Thus, KZFP-TE interactions may reflect not only reciprocal adaptation between mobile elements and their hosts, but also the long-term regulatory integration of TE-derived sequences into host genomes [13].

### 3.3. From Genomic Proximity to “Arm-in-Arm” Expansion of KZFPs and ERVs

The remarkable expansion of KZFP repertoires may be facilitated, at least in part, by their genomic organization. New KZFP genes frequently arise through segmental duplication, generating clusters containing multiple paralogous copies that can subsequently accumulate mutations and diversify [85]. The resulting gene clusters provide a genomic substrate in which multiple related KZFPs can evolve in parallel, potentially facilitating the emergence of new DNA-binding specificities.

Importantly, recently expanded KZFP gene clusters are themselves highly enriched in repetitive DNA, including TEs and particularly LTR/ERVs of similar evolutionary age to the KZFP cluster expansion (Figure 2). This repeat-rich environment further increases the structural complexity of these loci and has historically made them difficult to assemble and annotate accurately. Consequently, comparative analyses of KZFP clusters have long been hampered by gaps and errors in reference genome assemblies, limiting our ability to reconstruct their evolutionary histories.

Most of the evidence for an association between recent KZFP gene cluster expansion and ERV accumulation comes from mice, where species-specific KZFP cluster expansions are associated with the enrichment of mouse-specific ERVs (Figure 2, top). In primates, by contrast, we do not observe the same level of infiltration of the genome by new species-specific ERVs. Rather, primate genomes experienced an earlier wave of ERV infiltration at the clade level (Figure 1), and some KZFP gene clusters subsequently underwent primate-specific expansion and are enriched in primate-specific ERVs (Figure 2, bottom). Thus, the evolutionary timescale of KZFP and ERV expansion can differ between lineages, while the association between KZFP diversification and ERV accumulation may nevertheless reflect a conserved evolutionary process [81].

The increasing availability of long-read sequencing technologies, together with improved de novo genome assembly and pangenomic approaches, is now making these previously inaccessible genomic regions increasingly tractable. Comparisons across species, as well as between individuals and populations within species, are beginning to reveal the remarkable structural diversity of KZFP clusters and to provide new insight into the mechanisms underlying their expansion [81,86,87].

Recent work has highlighted the potential contribution of TEs, and ERVs in particular, to the structural evolution of KZFP clusters. New ERV integrations introduce additional repetitive sequences that can share sequence homology or microhomology with pre-existing repeats, increasing the potential for non-allelic homologous recombination and other rearrangements. Such events can generate substantial structural variation, including segmental duplications, deletions, and inversions. In young KZFP clusters, segmental duplications encompassing both KZFP genes and ERVs can therefore increase the copy number of both types of sequences simultaneously. Rather than acting solely as antagonistic partners evolving independently in response to one another, KZFPs and ERVs can thus become physically linked substrates of the same genomic duplication processes (Figure 3).

This mechanism provides a potential basis for an “arm-in-arm” mode of expansion: the same structural events that increase the number of KZFP copies can also increase the number of nearby ERV copies, generating additional genetic material upon which mutation, selection, and functional diversification can subsequently act. In this view, the evolutionary relationship between KZFPs and ERVs is not restricted to reciprocal adaptation at the level of sequence recognition. Their physical proximity within rapidly evolving genomic regions may itself contribute to the parallel expansion and diversification of both families.

### 3.4. KZFP Clusters as Genomic Environments for Retroviral Persistence and Diversification

The close physical association between KZFP clusters and ERVs raises a further, more speculative possibility: could KZFP-rich genomic regions influence not only the expansion of host defense genes, but also the fate of retroviral sequences that integrate within or near these loci? The available evidence suggests that KZFP clusters may have a dual role. On the one hand, they encode potent sequence-specific repressors that can rapidly silence newly integrated retroviral sequences; on the other hand, their repetitive and structurally dynamic genomic environment may provide conditions that favor the persistence and, in some circumstances, expansion of retroviral insertions. Whether these properties generally promote retroviral retention remains an open question, but several observations make the possibility worth considering.

One possibility is that some young KZFP clusters may represent relatively permissive genomic environments for newly integrated retroviral sequences. Several KZFP clusters contain genes with restricted or context-dependent expression and display repressive chromatin marks [76,88]. Retroviral insertions occurring within such regions may therefore be subject to strong silencing and consequently experience limited immediate deleterious effects on host gene expression. At the same time, the same silencing machinery could constrain the activity of the inserted retrovirus, reducing its potential to cause insertional damage or immune activation. Such a scenario could potentially facilitate the long-term retention of retroviral sequences.

Intriguingly, this possibility may extend beyond endogenous retroviruses to newly integrated sequences derived from exogenous retroviruses. In humans, analyses of HIV-1 integration sites have identified enrichment of proviral integrations in genomic regions associated with KRAB-ZFP genes, raising the possibility that KZFP clusters might represent safe harbors for the persistence of transcriptionally silent proviruses [89]. Likewise, in mice, some young KZFP clusters have been invaded by exogenous retroviruses, providing particularly interesting examples in which retroviral integrations and KZFP loci have subsequently evolved together. The Mouse Mammary Tumor Virus (MMTV), for example, likely integrated at a KZFP cluster at the distal end of mouse chromosome 4, with two ancestral integrations shared between *Mus musculus* and *Mus spretus*; subsequent expansion of the locus by segmental duplications encompassing one of these insertions in *Mus musculus*, enabled the expansion of MMTV copies as well, reaching 13 copies in the reference laboratory strain C57BL/6J [81].

The evolutionary consequences of such an arrangement are particularly intriguing because KZFP-mediated defense differs fundamentally from sequence-directed RNA-based defense systems. A retroviral insertion into a KZFP cluster does not directly provide the sequence information required to generate a KZFP capable of recognizing that retrovirus. Instead, the DNA-binding specificity of a new KZFP must arise through mutation and recombination of its zinc-finger array, followed by selection for variants capable of recognizing the invading element [90,91,92,93]. In contrast, in RNA-based defense systems like the piRNA pathway, insertion of a retroelement into a piRNA-producing locus can directly provide sequence information that is subsequently processed into small RNAs capable of targeting homologous elements elsewhere in the genome [94,95]. Thus, the physical association between a retrovirus and a protein-based defense locus may have very different evolutionary consequences from that between a retrovirus and an RNA-based defense locus.

The potential for TE-rich immune-gene clusters to facilitate the persistence and diversification of both host defense genes and their targets may not be unique to KZFPs. In plants, nucleotide-binding leucine-rich repeat (NLR) proteins constitute a major component of pathogen defense and are frequently organized in genomic clusters that undergo rapid evolution and structural rearrangement [96,97]. Several NLR clusters are enriched in TEs, including young LTR retroelements, which can contribute to local genomic instability and facilitate duplication and diversification of immune genes [98,99]. Although the molecular mechanisms and evolutionary histories of plant NLRs and mammalian KZFPs are distinct, these parallels raise the broader possibility that genomic regions encoding rapidly evolving protein-based defense systems may themselves become hotspots for TE accumulation and structural diversification.

Taken together, these observations suggest that KZFP clusters may have a dual evolutionary role. They function as genomic defense systems that detect and repress retroviral sequences, but their repetitive architecture and propensity for structural rearrangement may simultaneously create genomic environments in which retroviral insertions can persist and, potentially, expand. At present, the extent to which this represents a general mechanism rather than a consequence of local genomic history remains unclear. Although the current evidence is centered on KZFP and retroviral loci, the underlying principle may not be restricted to immune-gene clusters or retroviral sequences, as other rapidly evolving gene families and repetitive TEs could in principle experience similar structural coupling [100]. Nevertheless, considering KZFP clusters as both defensive loci and dynamic genomic environments for retroviral sequences broadens the classical arms-race framework and provides a potential mechanistic basis for the intertwined evolution of KZFPs and ERVs.

## 4. Conclusions and Future Perspectives

The increasing availability of complete, haplotype-resolved genomes and population-scale pangenomes now offers an unprecedented opportunity to investigate the structural evolution of repeat-rich regions, including KZFP gene clusters. Comparative analyses across closely related species and populations should help determine how frequently ERV-mediated recombination contributes to KZFP cluster expansion, how often KZFP and ERV copies are duplicated together, and whether such events are preferentially retained over evolutionary time.

Importantly, KZFP clusters are not the only genomic environments characterized by an abundance of repetitive DNA: TEs, including ERVs, are also extensively represented in centromeric and pericentromeric regions, which are among the most structurally dynamic and evolutionarily complex portions of many genomes beyond the mammalian lineage [101,102,103,104]. Understanding whether the shared repetitive nature of these genomic environments has convergent consequences for TE persistence, recombination, and structural evolution could provide a broader context for the proposed arm-in-arm model. Integrating comparative and population genomic approaches with functional assays will be particularly important to establish whether newly emerged KZFPs acquire regulatory activity against the retroelements accompanying their expansion, and whether retroviral integrations within KZFP clusters indeed have distinct evolutionary fates compared with integrations elsewhere in the genome. Newly endogenizing retroviruses and exogenous retroviral integrations could provide complementary systems in which some of these processes can be observed much closer to their origin.

## Figures and Tables

**Figure 1:**
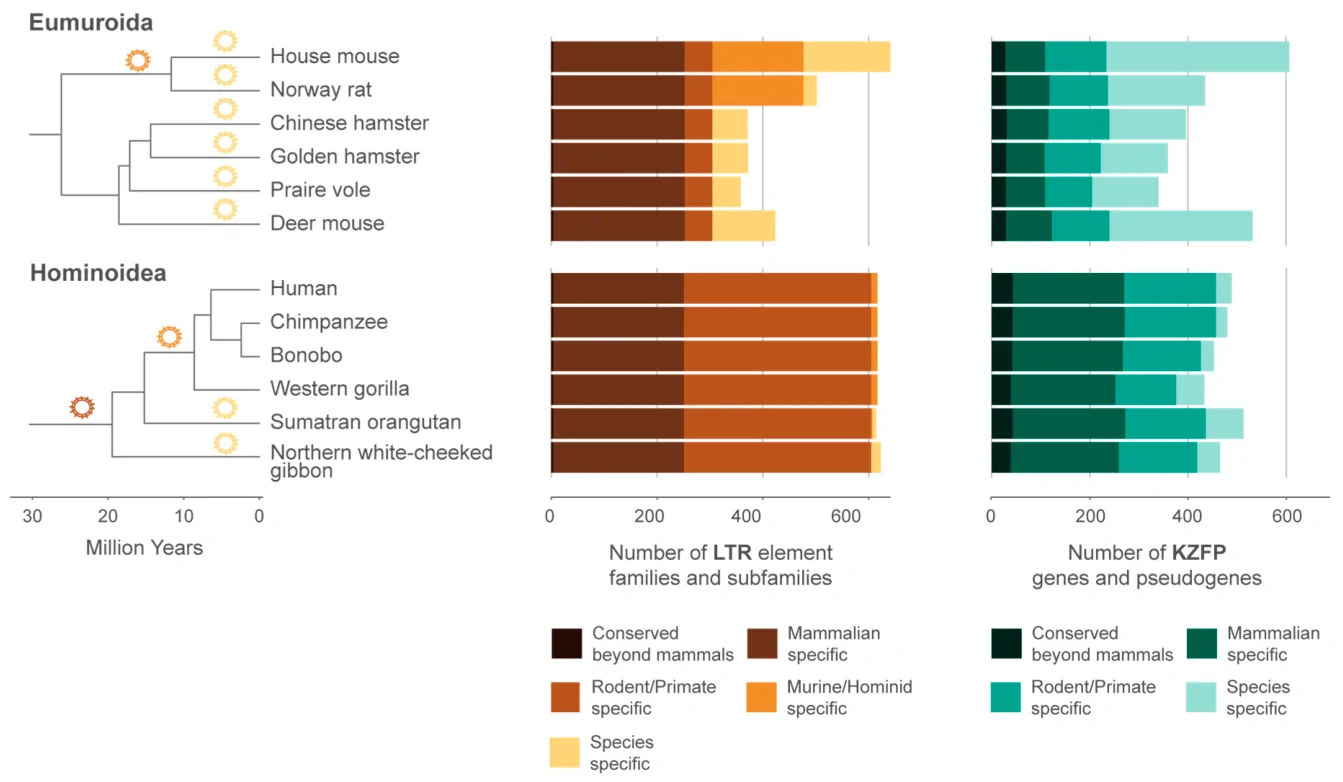
The heterogeneity of KZFP repertoires across example rodent and primate species mirrors the heterogeneity of the LTR elements emerging in those species. Only 4 families of LTR elements belong to the oldest “Conserved beyond mammals” category. The number of LTR element families and subfamilies was calculated based on FamDB V3; KZFP gene and pseudogene counts were calculated from Imbeault et al. 2017 [12]. Waves of retroviral infiltration are indicated by colored retrovirus-like symbols on the phylogenetic trees, with colors corresponding to the evolutionary categories shown in the legend.

**Figure 2:**
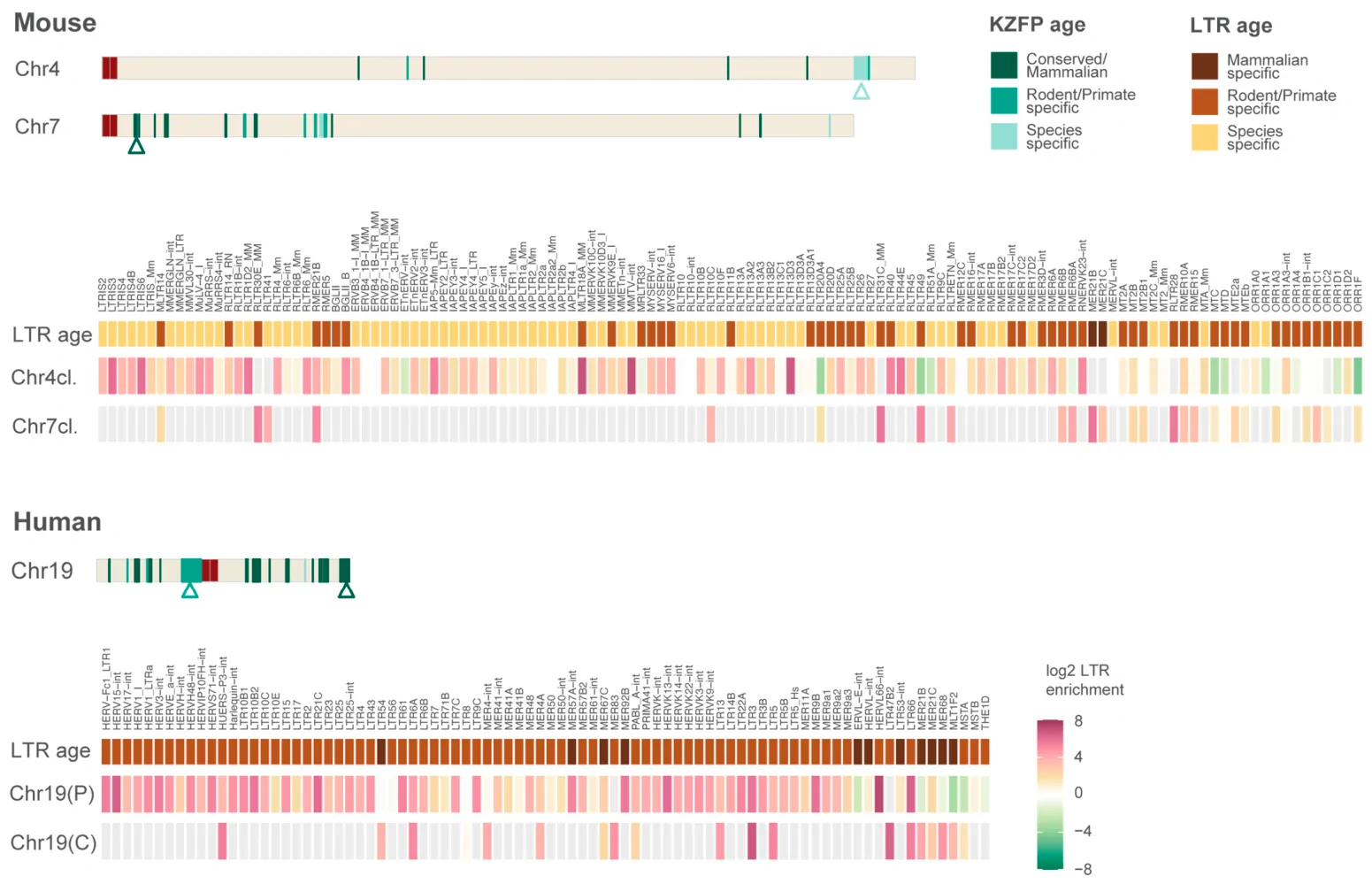
KZFP gene clusters are enriched for LTR/ERVs of similar evolutionary age. KZFP cluster distribution and their age is shown for mouse chromosomes 4 and 7, and for human chromosome 19. LTR/ERV enrichment is shown for clusters of different age (highlighted by a triangle at the bottom of the chromosomes). For human KZFP clusters, P and C indicate “primate” and “conserved”, respectively.

**Figure 3:**
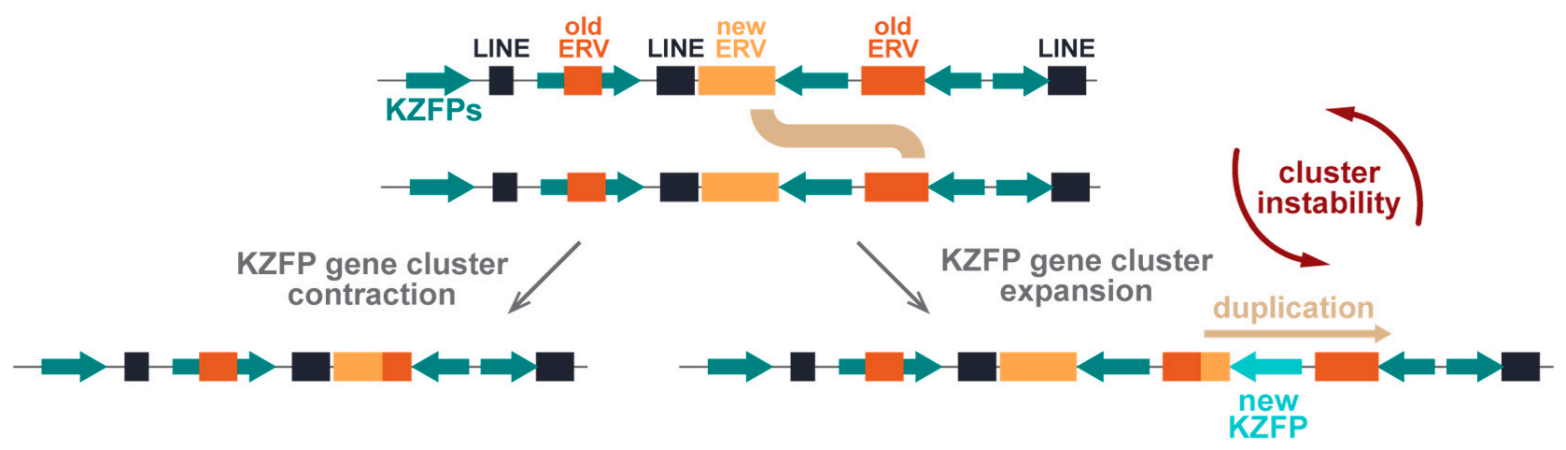
Proposed “arm-in-arm” model for simultaneous expansion of KZFP genes and ERVs.

## Data Availability

No new data were created in this study.
